# Immunoprofiling of Breast Cancer Antigens Using Antibodies Derived from Local Lymph Nodes

**DOI:** 10.3390/cancers11050682

**Published:** 2019-05-16

**Authors:** Anna Rachel Young, Jessica Da Gama Duarte, Rhiannon Coulson, Megan O’Brien, Siddhartha Deb, Alex Lopata, Andreas Behren, Suresh Mathivanan, Elgene Lim, Els Meeusen

**Affiliations:** 1 La Trobe Institute for Molecular Science (LIMS), La Trobe University, Melbourne 3086, Australia; a.young@latrobe.edu.au (A.R.Y.); s.mathivanan@latrobe.edu.au (S.M.); 2 Olivia Newton-John Cancer Research Institute, Level 5, ONJ Centre, Heidelberg, VIC 3084, Australia; Jessica.Duarte@onjcri.org.au (J.D.G.D.); megan.obrien@onjcri.org.au (M.O.); Andreas.Behren@onjcri.org.au (A.B.); 3 School of Cancer Medicine, La Trobe University, Melbourne 3086, Australia; 4 Garvan Institute of Medical Research, St Vincent’s Clinical School, Darlinghurst, NSW 2010, Australia; r.coulson@garvan.org.au (R.C.); e.lim@garvan.org.au (E.L.); 5 Consultant Pathologist, Anatpath. 120 Gardenvale Rd, Gardenvale Melbourne 3185, Australia; deb_siddhartha@hotmail.com; 6 CancerProbe Pty Ltd., P.O. Box 2237, Prahran 3181, Australia; alex.lopata15@gmail.com

**Keywords:** tumor antigen, immune profile, lymph node, antibody secreting cell, biomarker, microarray, breast cancer, immunotherapy

## Abstract

Tumor antigens are responsible for initiating an immune response in cancer patients, and their identification may provide new biomarkers for cancer diagnosis and targets for immunotherapy. The general use of serum antibodies to identify tumor antigens has several drawbacks, including dilution, complex formation, and background reactivity. In this study, antibodies were generated from antibody-secreting cells (ASC) present in tumor-draining lymph nodes of 20 breast cancer patients (ASC-probes) and were used to screen breast cancer cell lines and protein microarrays. Half of the ASC-probes reacted strongly against extracts of the MCF-7 breast cancer cell line, but each with a distinct antigen recognition profile. Three of the positive ASC-probes reacted differentially with recombinant antigens on a microarray containing cancer-related proteins. The results of this study show that lymph node-derived ASC-probes provide a highly specific source of tumor-specific antibodies. Each breast cancer patient reacts with a different antibody profile which indicates that targeted immunotherapies may need to be personalized for individual patients. Focused microarrays in combination with ASC-probes may be useful in providing immune profiles and identifying tumor antigens of individual cancer patients.

## 1. Introduction

Immunotherapy has changed the landscape of cancer treatment for an increasing number of solid tumors and has increased interest in the immunological status of cancer patients [1,2]. An antitumor immune response, generated naturally or through vaccination, is critically dependent on the presence of tumor antigens generated during cancer formation. Tumor antigens expressed on the surface of tumor cells can also provide attractive targets for new and personalized immunotherapy approaches [3,4]. A major effort is therefore being directed towards identifying tumor antigens expressed by individual cancer patients.

Tumor antigens are recognized by both T and B cell receptors, although in different formats (peptide vs. native molecule), and most B cell responses require T cell help for clonal expansion and the generation of antibody-secreting cells (ASCs) [5]. The initial interactions between antigen-presenting cells and T and B cells takes place in the lymph nodes draining the target tissues and result in the production of antigen-specific ASCs. Fully differentiated ASCs can secrete thousands of antibody molecules per second [6] and therefore provide a significant biological amplification of molecular changes occurring in cancer cells and a reflection of the tumor’s antigenic profile. Antibodies produced by ASCs are released into the blood stream, where they are vastly diluted with nonspecific antibodies. The formation of immune complexes can also reduce sensitivity and specificity when serum is used as the antibody source in immunoassays [7,8]. Recent advances in protein microarray-based antibody profiling have in part addressed these issues with reported high sensitivity and reproducibility [9]. Nonetheless, additional improvements may be obtained by selecting a more specific source of antitumor antibodies [4].

It has been known for a long time that regional lymph nodes of breast tumors are often enlarged, even in the absence of metastatic tumor cells, indicating that an active immune response is taking place [10]. Changes in the general immune profile of breast cancer-draining lymph nodes have also been found to predict recurrence regardless of metastasis status [11]. More recently, tumor-draining lymph nodes of breast cancer patients have been shown to contain increased numbers of germinal centers and immunoglobulin (Ig)G^+^ B cells [12,13] and the clonality and diversity of B cell receptor genes have been correlated with survival and response to immunotherapy in subtypes of breast and ovarian cancer [14]. These findings indicate that capturing the antibody response of tumor-specific ASCs in the lymph node of individual patients may provide a more specific source of antibodies for identifying tumor antigens and a patient’s baseline immune status and antigen profile.

Previous infectious disease studies have shown that short term culture of lymph node cells results in the release of antibodies in the culture supernatant that reflect the total antigen repertoire of the pathogen present in the target tissue at the time of lymph node collection [15,16]. The culture supernatants, containing antibodies secreted by in vivo induced ASCs, termed ASC-probes, have been used to identify stage-specific pathogen antigens using standard immune-technological approaches [15,17]. In the present study, we apply this technology to generate ASC-probes from the lymph nodes of breast cancer patients and compare the individual antibody response against breast cancer cell lines. In addition, we combine the ASC approach with microarray screening to illustrate a rapid method for tumor-specific antibody profiling and antigen identification.

## 2. Results

### 2.1. Immunoglobulin Concentration and Antibody Reactivity Profile of ASC-Probes against MCF-7 Extracts

ASC-probes were generated from the lymph nodes of 20 breast cancer patients, whose characteristics are presented in Table 1. The mean concentration of IgG in the ASC-probes, excluding patients 3 and 6, was 0.513 µg/mL (Table 2). ASC-probes obtained from patients 3 and 6 were significantly higher at 3.6 µg/mL and 5.9 µg/mL, respectively. The lymph node processed for ASC-probe 3 was macroscopically assessed to contain a tumor, and ASC-probe 6 was produced from a pool of large lymph nodes from the only patient with occult primary cancer.

**Figure 1:**
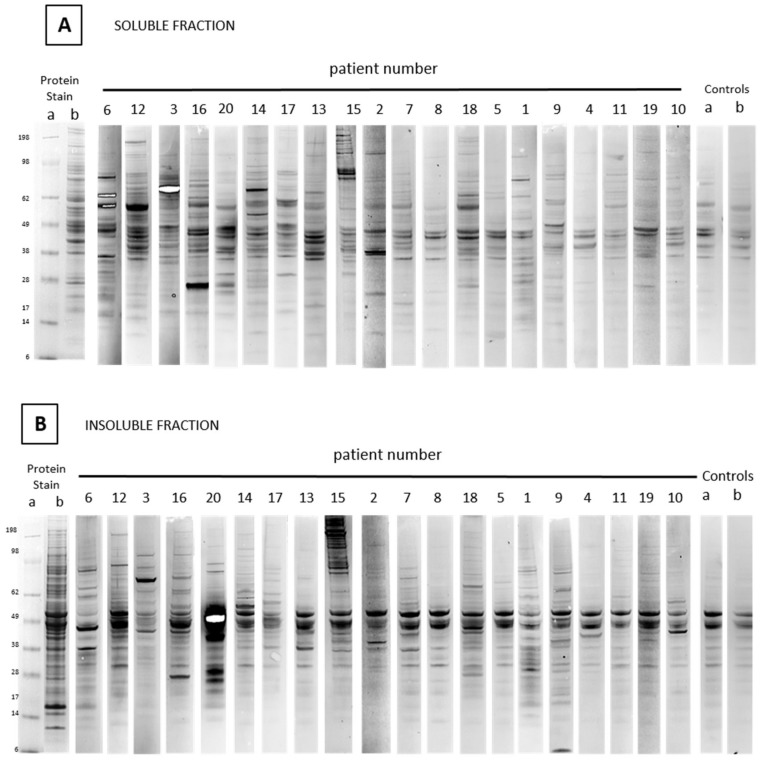
One-dimensional (1D) Western blots of soluble (**A**) and insoluble (**B**) fractions of MCF-7 cell extracts screened with antibody-secreting cell (ASC)-probes from 20 breast cancer patients. Molecular weight markers on the left are recorded from prestained markers (a) and the adjacent lane (b) shows the coomassie stained cell extract. Control lanes on the right are screened with serum from healthy women (a) or secondary antibody conjugate only (b). White bands are the result of extremely high levels of localized signal.

Individual one-dimensional (1D) Western blots of ASC-probes against soluble or insoluble extracts of MCF-7 cells were adjusted for maximum definition and are shown in Figure 1. Interestingly, all reactive ASC-probes showed a unique pattern of band reactivity, although some bands appear shared amongst several samples. The strong doublet band around 60 kD that appeared across all blots in the insoluble fraction, including the negative control serum, was identified as keratin 8 type II and keratin 18 type I by mass spectrometry (not shown).

Relative reactivity levels of each ASC-probe were determined against negative controls by density quantification of Western blots and are shown in Table 2. Ten ASC-probes showed strong overall reactivity with MCF-7 cell extract, while the remaining 10 reacted weakly or not at all (Figure 1 and Table 2). Reactivity levels were generally higher for the soluble than insoluble fractions, except for ASC-probes 20 and 15 (ratios 0.5 and 0.4, Table 2), both derived from patients diagnosed with a multifocal tumor.

### 2.2. Comparative Reactivity of ASC-Probes against Different Breast Cancer Cell Lines

**Figure 2:**
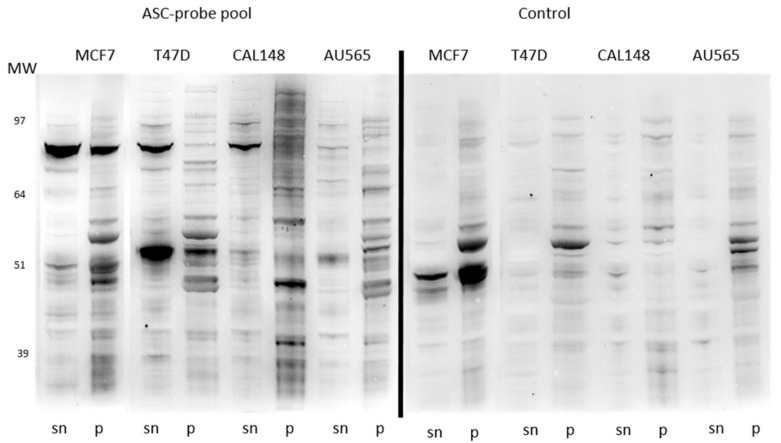
1D Western blots of soluble (sn) and insoluble (p) extracts of different cancer cell lines screened with a pool of antibody-secreting cell (ASC)-probes (**left panel**) or control serum (**right panel**).

A pool of four strongly reacting ASC-probes was screened against different breast cancer cell lines (Figure 2). While some reactive bands were common between some of the cell lines, others were distinct. In particular, the strong band at approximately 80 kDa in MCF7 extract was not obvious in extracts from AU565 cells, while a new strong band appeared around 50 kDa in soluble T47D extract which was not prominent in any of the other cell lines.

### 2.3. Antigen Recognition Profile of ASC-Probes on a Custom Protein Microarray

**Figure 3:**
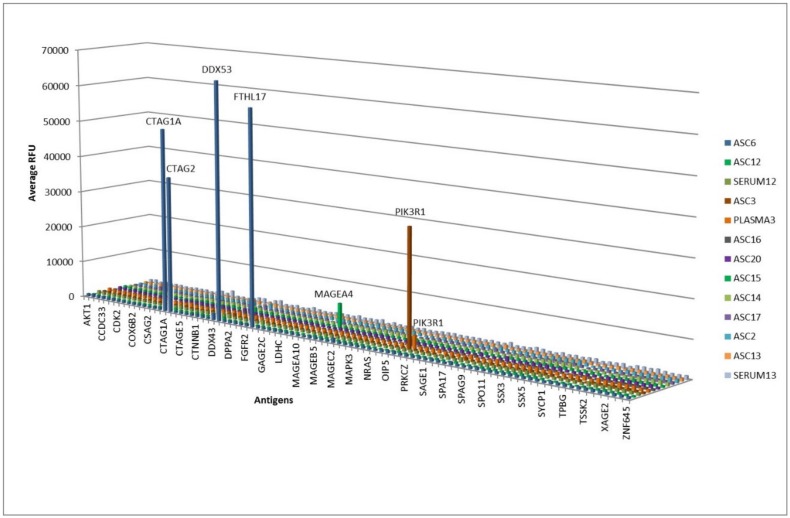
Microarray screening of 10 antibody-secreting cell (ASC)-probes and 3 matching sera/plasma.

Antibody profiling using a custom protein microarray showed low to high (<5000 to >30,000 relative fluorescence units) cancer antigen-specific antibodies in 3/10 positive ASC-probes (Figure 3). Antigen specificities included members of the cancer-testis antigen (CTAG) family, such as CTAG1 (also known as NY-ESO-1), CTAG2, DEAD-box helicase 53 (DDX53), ferritin heavy polypeptide-like 17 (FTHL17) (ASC#6) and melanoma-associated antigen 4 (MAGEA4) (ASC#15), as well as Phosphoinositide-3-Kinase Regulatory Subunit 1 (PIK3R1) (ASC#3). Of these, plasma was collected at lymph node resection for one patient (#3), and an overlapping antibody profile was seen, but at much lower antibody titer (10-fold lower).

## 3. Discussion

Tumor antigens interact with both T and B cell receptors in the lymph nodes draining the affected tissue, resulting in B cell differentiation and production of antigen-specific antibodies. In the present study, we demonstrate the capture of these antibodies by the short-term culture of lymph node cells and harvesting of the supernatant (ASC-probes), containing secreted antibodies. As only recently stimulated and differentiated B cells secrete antibodies, there is no need for purification of ASCs away from unstimulated B cells or other non-antibody producing leukocytes, thereby significantly simplifying the process of producing specific antibody-probes. Previous studies have attempted to obtain tumor-specific antibodies from tumor-draining lymph nodes by recombinant VH single chain antibody production or by immortalizing B cells [12,18]. However, these are time-consuming and selective processes. Similarly, the transplantation of tumor tissue containing B cells into immune-deficient mice has demonstrated the restricted specificity of antibodies produced by these B cells; however, the procedure is time-consuming and requires specialized resources [19]. Ex vivo cultures of purified B cells have also been generated from tumor biopsies [20], and tertiary lymphoid structures (TLS) present in the lungs of some cancer patients and the supernatant antibodies were shown to have specific antitumor reactivity [21,22]. These procedures require adequate tissue and complex cell isolation procedures, and tissue B cells and TLS are not present in all patients or tumor types. By contrast, the lymph nodes that drain solid tumors, are easy to process without in vitro manipulation, and the clonal and functional relationship between antibodies in tissues and draining lymph nodes has previously been demonstrated [23,24]. Further improvements to the ASC-probe approach may be achieved by the direct labeling of lymph node derived antibodies, and blocking using control human sera to detect and identify tumor antigens on cancer and control breast tissue.

Breast cancer is generally considered to be poorly immunogenic, in particular the estrogen and progesterone positive subtype [25,26] which was the predominant subtype in this study (70%). However, half of the ASC-probes reacted strongly against extracts of the breast cancer cell line MCF-7 on Western blots. Several ASC-probes that did not react or reacted only weakly with MCF-7 extracts had higher IgG concentrations than the strong reactors, suggesting they may also have antitumor reactivity but against antigenic targets not present in MCF-7 cells. Cell lines are unlikely to represent the full antigenic repertoire of the original tumor [27], and different cell lines may display different tumor antigens, as demonstrated by the variation in Western blot reactivity against four breast cancer cell lines in the present study. Overall, this suggests that most patients were able to generate an antitumor immune response. Interestingly, each positive ASC-probe reacted with a distinct recognition profile in both the Western blot and microarray assay. It is well recognized that there is a large molecular heterogeneity in breast tumors [28], and the present results show that this is also reflected in the individual antibody reactivity profile of each patient. Although the antigen binding patterns are heterogeneous, further examination of ASC-probes derived from larger patient cohorts may reveal similarities between specific antigens and antibody isotype profiles in patient subtypes.

A protective immune response against a tumor is thought to be directed against tumor antigens presented on the surface of cancer cells, either through killing by cytotoxic T cells or antibody-mediated cellular cytotoxicity. By contrast, soluble antigens may have immunosuppressive effects [12]. Membrane-bound antigens are generally present in the insoluble cell fraction, and the present study found considerable variation in the relative response to soluble and insoluble antigens, which may determine the overall effectiveness of the immune response generated.

Screening of antibodies on protein microarrays allows direct identification of the reactive protein. Several protein arrays are now available commercially with a varying number and quality of recombinant proteins (reviewed in [9]). A custom microarray, containing predominantly cancer-testis (CT) antigens, was available and used to screen all strongly-reactive ASC-probes. CT antigens have normal expression restricted to germ cells but can be aberrantly expressed in tumors [29]. Screening of ASC-probes on this custom array identified antigen reactivity in 3/10 samples. One ASC-probe (#6) showed high reactivity against 4 distinct CT antigens, products from the genes CTAG1, CTAG2, DDX53, and FTHL17. This ASC-probe was derived from the only patient with a hormone negative and an equivocal (2+) human epidermal growth factor receptor 2 (HER2) score. This subtype (as well as the triple negative subtype) is known to be highly proliferative, and the tumor’s overexpression of immune-related genes has been shown to correlate with a better prognosis, suggesting a possible role for immunotherapy in this subgroup [25,30]. Another ASC-probe (#3) derived from a tumor-containing lymph node reacted strongly with a ~80 kDa protein in the soluble MCF-7 extract, and with the product of the PIK3R1 gene on the microarray. The PIK3R1 protein was also detected in the semipurified MCF-7 fraction by mass spectrometry (not shown). The PIK3R1 gene product is a protein kinase regulator with expected molecular weight of 84kDa known to be involved in tumor cell migration and invasion [31]. Mutations in PIK3R1 have been found in breast cancer [32] and may result in the generation of a neoantigen. As far as we know, no natural antibody response has previously been reported against this molecule. Matching plasma from this patient also reacted with the PIK3R1 protein on the microarray, although at a much lower level. This is in agreement with a recent study using next-generation sequencing and proteomics to demonstrate a link between affinity-matured expanded B-cell clones in tumor-draining lymph nodes and antitumor Ig in the blood [33]. In view of a more than 15-fold higher IgG concentration expected in the plasma sample [34] compared to the ASC-sample, the much lower (10×) plasma reactivity confirms that antibodies produced by lymph node ASCs are vastly diluted when released into the bloodstream. A third ASC-probe (#15) reacted with the CT antigen, melanoma-associated antigen 4 (MAGEA4). While several studies have identified the presence of MAGEA4 protein in breast cancer, this antigen was not detected with serum of breast cancer patients after extensive screening of cDNA libraries containing the recombinant protein [12], possibly due to masking by background reactivity or immune complex formation.

The absence of antibody reactivity detected in 7/10 strongly reacting ASC-probes on the microarray is likely due to its restricted cancer antigen content. Nonetheless, the high sensitivity and minimal sample volume (0.1 mL of ASC-probe) required for protein microarray assays make this an attractive method to determine antigen specificity. Larger microarrays containing almost the entire human proteome are now available commercially [9] and could be used in future studies in combination with tumor-specific ASC-probes to select a panel of reactive breast cancer antigens for further validation as potential biomarkers for diagnosis, or targets for personalized immunotherapies, such as cancer vaccines and chimeric antigen receptor (CAR)-T cells. Neoantigens, defined by mutational studies, have also been shown to have a high predictive value for response to treatment [3], and a specific antibody fingerprint may aid in selecting patients that would benefit from specific immunotherapies.

## 4. Materials and Methods

### 4.1. Generation of Lymph Node ASC-Probes and Quantitation of Secreted Antibodies

ASC-probes were generated from the lymph nodes removed during surgery of 20 patients with breast cancer, under the Austin Health Human Ethics Research Committee approved protocol H2012-04446, and St Vincent’s Hospital Human Ethics Research Committee approved protocol HREC/16/SVH/29. Data on estrogen receptor (ER), progesterone receptor (PR), and human epidermal growth factor receptor 2 (HER2) expression of the tumors were collected (Table 1). Single cell suspensions were prepared from lymph node tissue using published techniques [15] and cultured in vitro for 5 days with 0.1 ng/mL recombinant IL-6 (R&D Systems, Minneapolis, MN, USA). Cell culture medium containing secreted antibodies was harvested after centrifugation to remove lymphocytes (=ASC-probes). Total IgG secreted into the culture medium was measured using a human IgG quantification kit (FastElisa; R&D Biotech, Besancon, France).

### 4.2. Western Blotting of ASC-Probes against Soluble and Insoluble Extracts of Cancer Cell Lines

The breast cancer cell lines MCF7, T47D, CAL148, and AU565 were maintained in RPMI 1640 medium supplemented with 10% fetal calf serum (FCS). Soluble and insoluble cell extracts were prepared from cells grown to >80% confluence and washed twice in phosphate buffered saline (PBS) to remove FCS. They were then put into lysis buffer, 5 mM tris pH 7.4 with protease inhibitors (1% protease inhibitor cocktail, Roche), and frozen until required. The cells were disrupted by sonication and the soluble cell extract collected following centrifugation at 16,000 *g* for 15 min at 4 °C. The remaining insoluble material was washed with lysis buffer containing DNase (25 U/mL, ThermoFisher, Waltham, MA USA) with gentle agitation for 45 min at 4 °C and centrifuged as before. The pellet was resuspended in 50 mM tris pH 6.8, 10% glycerol, 2% sodium dodecyl sulfate (SDS) with protease inhibitors, by aspiration 5 times through a 27-gauge needle, to shear remaining DNA.

To test the antibody reactivity of ASC-probes against MCF-7 extracts, the samples were diluted in reducing sample buffer to a final concentration of approximately 16 µg protein per lane, run on NuPAGE 4–12% Bis-tris precast gels (ThermoFisher, Waltham, MA, USA) with SeeBlue plus 2 prestained molecular weight markers and blotted to polyvinylidene difluoride (PVDF) membrane using an iblot (ThermoFisher, Waltham, MA, USA). The blots were blocked with 0.5% tween-20 PBS, for 1 h at room temperature, washed with 0.1% tween-20 in PBS (PBS-T), and incubated overnight at 4 °C with primary antibody, either ASC-probes diluted 1:2 in PBS-T, or control serum from healthy women diluted to 2–4 µg/mL IgG (in a solution of equal parts RPMI media with 10% FCS and PBS-T). Following washing, the blots were incubated with an horseradish peroxidase (HRP)-conjugated antihuman IgG (H&L) antibody (Abcam, Cambridge, UK, 1:40,000 in PBS-T) for 1 h at room temperature, washed again, and then developed using SuperSignal™ West Pico PLUS Chemiluminescent Substrate (ThermoFisher, Waltham, MA, USA).

For the analysis of ASC-probes against different breast cancer cell lines, the cell extracts were first separated using NuPAGE™ 10% Bis-tris precast gels, and blots screened with a pool of ASC-probes from patients 6, 3, 14, 13 at a 1:2 dilution and control serum as before.

### 4.3. Analysis of Overall Reactivity with Image J

Profile plots were generated for each 1D Western blot lane using Image J software (NIH, Madison, WI, USA) and a baseline drawn to enclose all the peaks within the area of reactivity. The area under the peaks was then quantitated for each ASC-probe using the Image J wand tool and divided by the area of a control lane exposed for a similar time, to obtain the relative reactivity ratio for each ASC-probe.

### 4.4. Antibody Profiling Using a Custom Protein Microarray

The ten strongest reacting ASC-probes and matching serum/plasma where available (n = 3) were screened using a custom cancer-specific protein microarray. Pooled sera from 1500–3000 healthy individuals were also assayed to determine cancer-specific thresholds. The array content was selected from the Immunome Protein Array list (Sengenics Corporation, Singapore), and consisted of 99 biotinylated full-length, correctly folded and functional cancer antigens (mainly cancer-testis (CT) antigens) (Supplementary Table S1). These were printed in triplicate onto streptavidin-coated glass HS slides (Schott, Jena, Germany) in 8-plex (8 replica arrays per slide) by Applied Microarrays and stored at −30 °C. After equilibration to room temperature, slides were blocked using free biotin to reduce nonspecific binding. Individual arrays were isolated using ProPlate multiwell chambers (Grace Bio-Labs, Bend, OR, USA) and incubated with a unique ASC-probe (1:2 dilution in 0.1% tween-20 PBS (PBST)) or serum (1:800 dilution in PBST)/plasma (1:400 dilution in PBST) sample, followed by a fluorescently-labeled antihuman IgG detection antibody (Invitrogen, Carlsbad, CA, USA). Arrays were then scanned using a GenePix 4000B microarray scanner (Molecular Devices, San Jose, CA, USA) at fixed gain settings, and the resulting data extrapolated with the GenePix Pro 7 software (Molecular Devices). Raw data were processed using the Protein Microarray Analyser software [35]. Cancer-specific antibody titers were classified as absent (below threshold), low (<5000 relative fluorescence units (RFU)), medium (5000–30,000 RFU) or high (>30,000 RFU) for each sample.

## 5. Conclusions

Lymph nodes draining solid tumors contain antibody-secreting cells (ASCs) that are generated in response to antigens expressed by the tumor. The short-term in vitro culture of such lymph node cells allows the capture of antibodies secreted by these in vivo induced ASCs and provides a unique, tumor-specific antibody probe containing the full polyclonal response against the tumor. Using ASC-probes from breast cancer patients revealed that most patients induced an immune response against tumor antigens, each with an individual antigen recognition profile. Lymph node-derived ASC-probes can be used to rapidly identify tumor antigens and immune profiles and may deliver new biomarkers and targets for both diagnosis and personalized immunotherapy. Further studies using larger patient cohorts are necessary to assess if lymph node-derived antibodies are correlated to disease stage and breast cancer subtypes.

## Figures and Tables

**Table 1:** Clinical characteristics of patients.

Patient No.	Age	Tumor Grade	ER	PR	HER2 (ISH)	TNM Classification	Tumor Size (mm)	Pathological Features
1	67	2	+	+	neg	T2N1M0	33	IDC
2	49	1	+	+	neg	T2N2M0	26	Invasive micropapillary carcinoma
3	42	3	+	+	2+ Non-amplified	T2N2M0	28	IDC
4	44	3	neg	neg	3+ Amplified	T3N2M0	65	IDC
5	66	3	neg	neg	3+ Amplified	T1N1M0	15 and 4.5	IDC, multifocal
6	58	3	neg	neg	2+ Amplified	TXN2M0	NA	Occult Primary Breast Cancer
7	73	2	+	+	neg	T3N2M1	110	IDC
8	72	3	+	+	neg	T2N1M0	22	IDC
9	78	2	+	+	neg	TXN3M0	NA	ILC
10	44	2	+	+	neg	T4N3M0	70	IDC, multifocal
11	58	1	+	+	neg	T2N1M0	25	IDC
12	60	2	+	+	neg	T3N2M0	60	IDC
13	63	3	+	+	2+ Amplified	T1N1M0	13	IDC
14	71	3	+	+	neg	T4N2M0	60	ILC
15	64	3	+	+	neg	T2N2M0	65 and 13	IDC, multifocal
16	45	3	+	+	neg	T2N2M0	25 and 15	IDC, multifocal
17	39	3	neg	neg	3+ Amplified	NA	4 (DCIS)	Residual DCIS post preoperative chemotherapy. Pathological complete response.
18	60	3	+	neg	3+ Amplified	T2N1M0	40	IDC
19	39	3	NA	NA	NA	T2N1M0	32	IDC
20	75	2	+	+	neg	T2N2M0	21 + 15	IDC, multifocal

Abbreviations: ER: estrogen receptor, PR: progesterone receptor, HER2: human epidermal growth factor receptor 2, IDC: Invasive ductal carcinoma, ILC: Infiltrating lobular carcinoma, ISH: In situ hybridization; NA: Not available.

**Table 2:** IgG concentration and relative strength of reactivity of ASC-probes of 20 breast cancer patients.

Patient Number	µg IgG/mL	Relative Reactivity Ratio ASC-Probe/Control ^1^		
		Soluble (S)	Insoluble (I)	Total (Ratio S/I)
		Strong		
6	5.882	12.69	11.54	24.23 (1.2)
12	0.902	15.82	3.34	19.16 (4.7)
3	3.6	12.33	6.58	18.92 (1.9)
16	0.453	5.52	3.20	8.72 (1.7)
20	1.150	2.29	5.04	7.34 (0.5)
15	0.396	1.44	3.21	4.66 (0.4)
14	0.341	3.90	2.04	5.94 (1.9)
17	0.790	3.59	1.60	5.20 (2.2)
2	0.468	2.47	1.71	4.18 (1.4)
13	0.423	3.26	1.77	5.03 (1.8)
Weak/negative				
7	0.549	1.85	1.44	3.29 (1.3)
8	0.421	2.10	1.35	3.45 (1.6)
18	0.760	1.53	1.04	2.57 (1.5)
1	0.174	0.79	1.20	1.99 (0.7)
10	0.207	0.62	0.66	1.28 (0.9)
5	0.173	0.8	1.3	2.2 (0.6)
19	1.060	0.75	0.75	1.50 (1.0)
4	0.188	0.9	0.7	1.6 (1.3)
9	0.539	0.91	0.89	1.80 (1.0)
11	0.912	0.87	0.68	1.55 (1.3)

^1^ Relative reactivity was determined by density scans of individual antibody-secreting cell (ASC)-probe Western blots against MCF-7 soluble or insoluble cell extract, divided by similar control blots.

## Supplementary Materials

The following are available online at https://www.mdpi.com/2072-6694/11/5/682/s1, Table S1: Custom protein microarray antigen content.
