# Supplementary material for: Immunoprofiling of Breast Cancer Antigens Using Antibodies Derived from Local Lymph Nodes
Source: Cancers (Basel). 2019 May 16;11(5):682. doi: 10.3390/cancers11050682 (PMC6562983; doi:10.3390/cancers11050682)
Supplement: Supplementary file 1 [file cancers-11-00682-s001.pdf]

Supplementary materials

Table S1. Custom protein microarray antigen content.

**AKT1**	**DDX43**	**MAGEC3**	**SSX1**
AXL_int	DDX53	MAPK1	SSX2
BAGE2	DKKL1	MAPK3	SSX3
CCDC33	DPPA2	MLANA	SSX4
CCDC36	EGFR_ext	NLRP4	SSX4B
CDC25A	FES	NRAS	SSX5
CDK2	FGFR2	NXF2	SSX7
CDK4	FTHL17	NXF2B	SYCE1
CEACAM1	GAGE1	OIP5	SYCP1
COX6B2	GAGE2C	PIK3R1	THEG
CREB1	HORMAD1	POU5F1	TP53
CSAG1	KRAS	PRKCZ	TPBG
CSAG2	LDHC	PTEN	TPTE
CT45A1	LEMD1	ROPN1	TSGA10
CT47A1	LIPI	SAGE1	TSSK1B
CT62	MAGEA10	SOX10	TSSK2
CTAG1A	MAGEA4	SOX2	TSSK3
CTAG2	MAGEB1	SPA17	TSSK6
CTAGE1	MAGEB2	SPACA3	TYR
CTAGE5	MAGEB3	SPAG4	XAGE1D
CTCFL	MAGEB4	SPAG9	XAGE2
CTNNA2	MAGEB5	SPANXN1	XAGE4
CTNNB1	MAGEB6	SPANXN2	ZNF165
CXorf48	MAGEC1	SPANXN4	ZNF645
Cxorf61	MAGEC2	SPO11	
